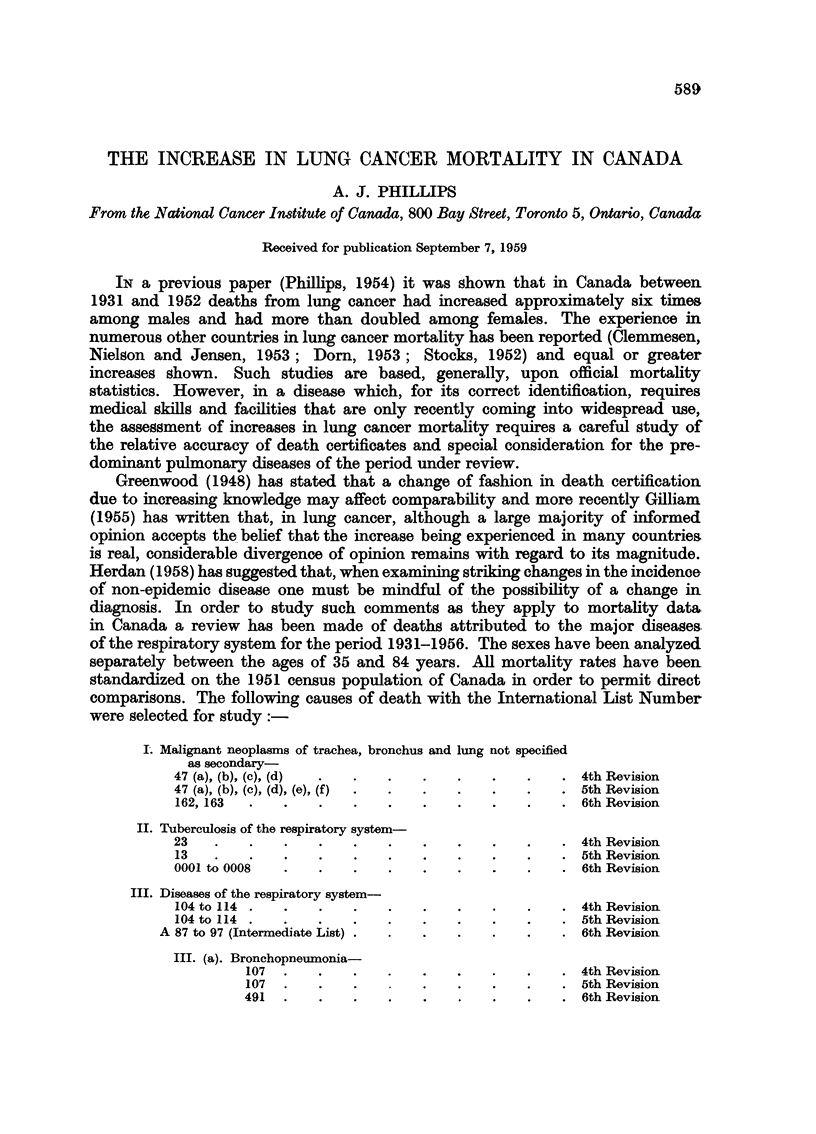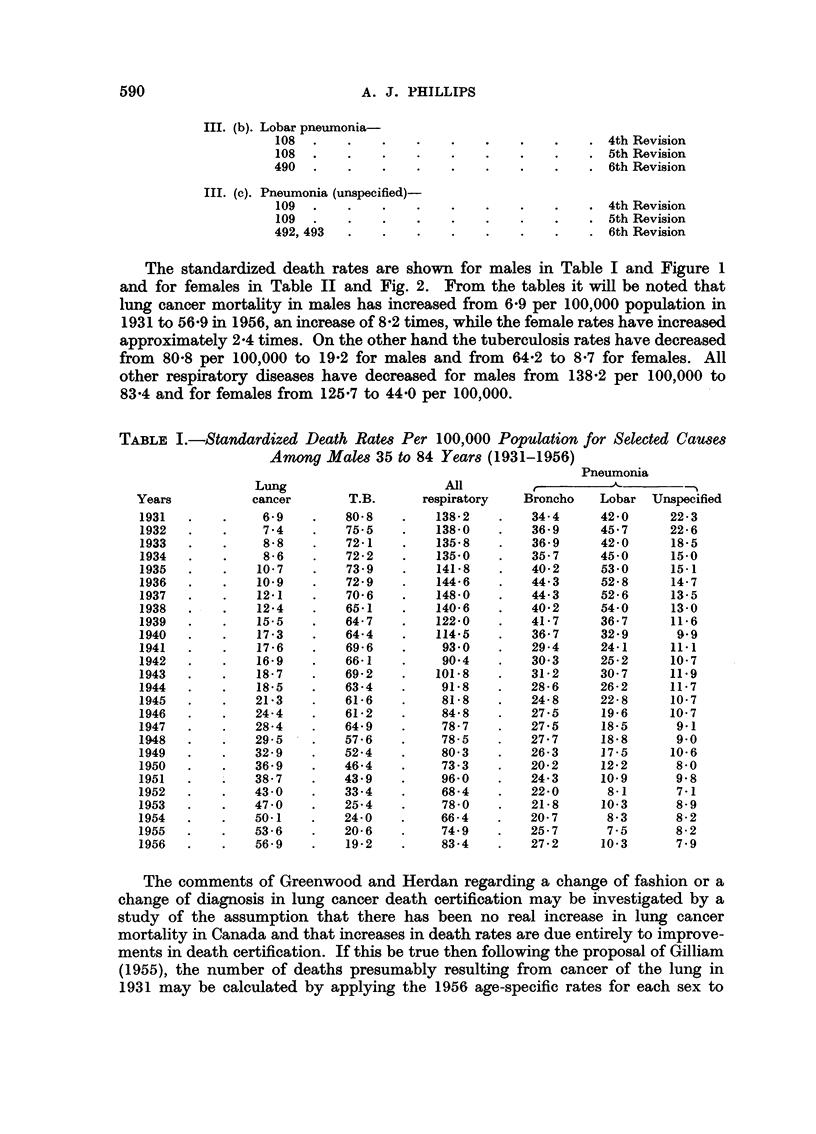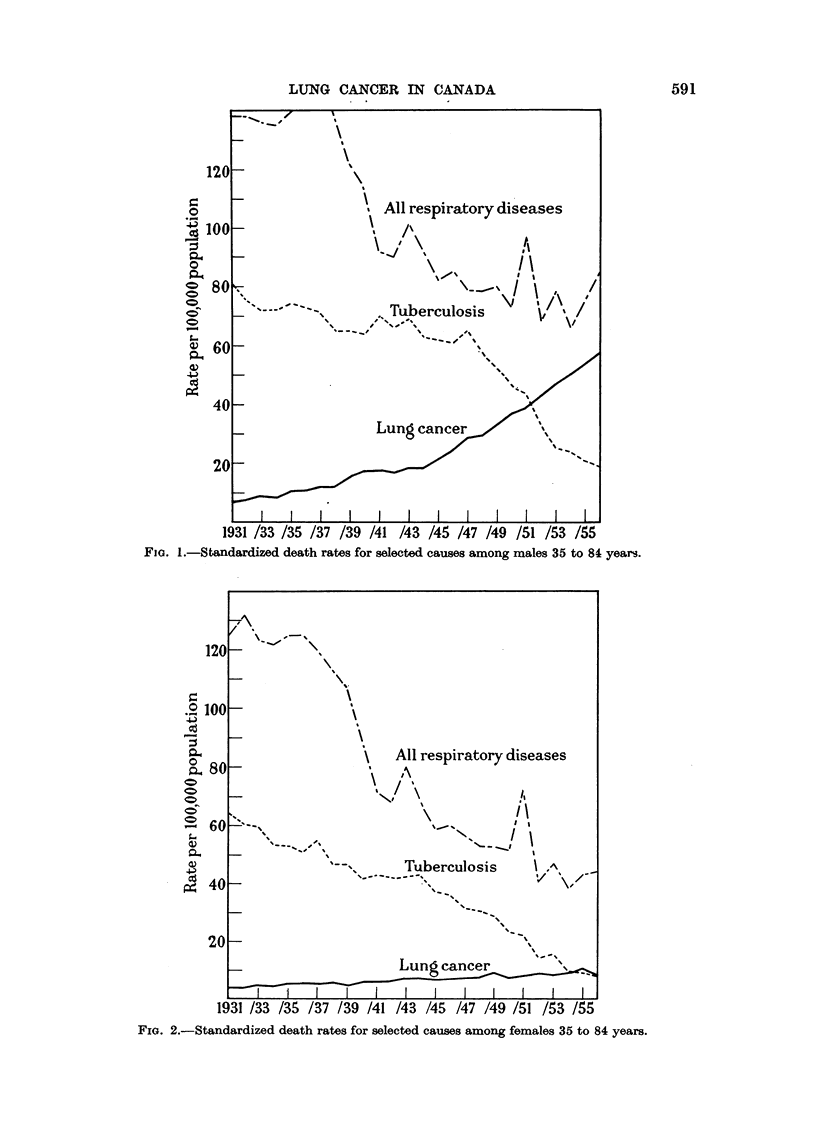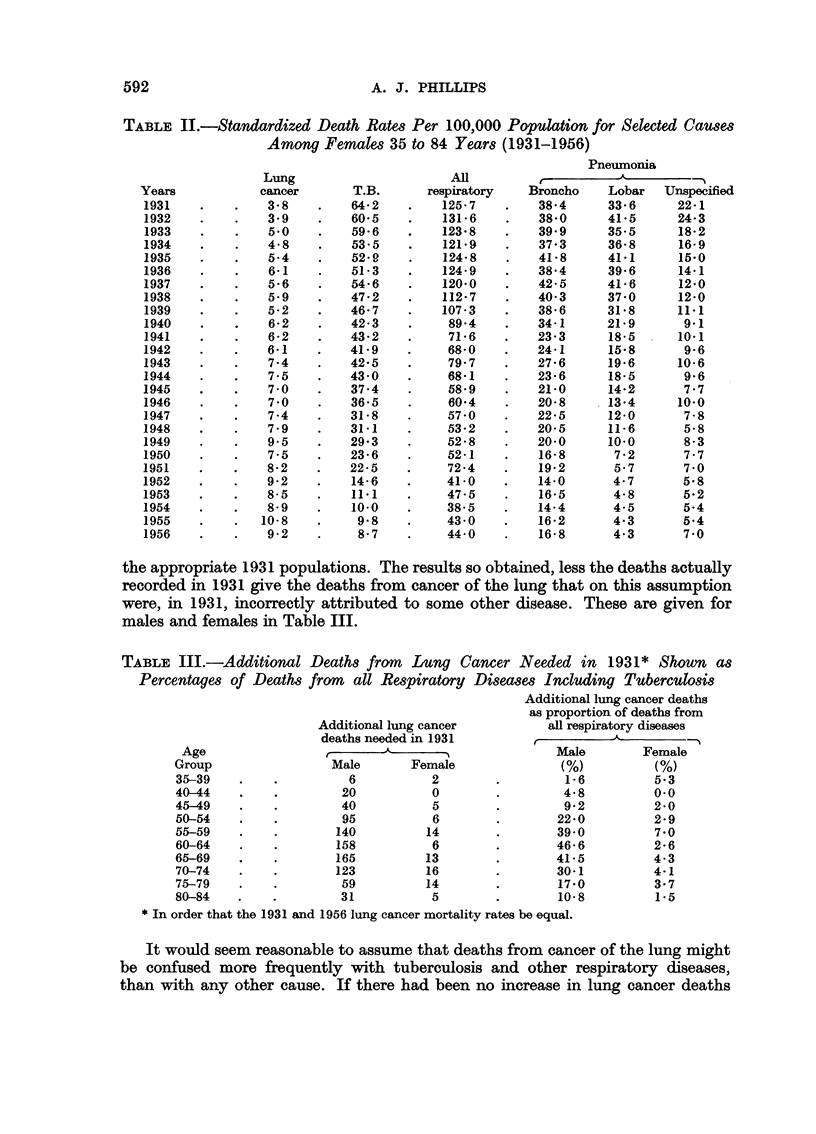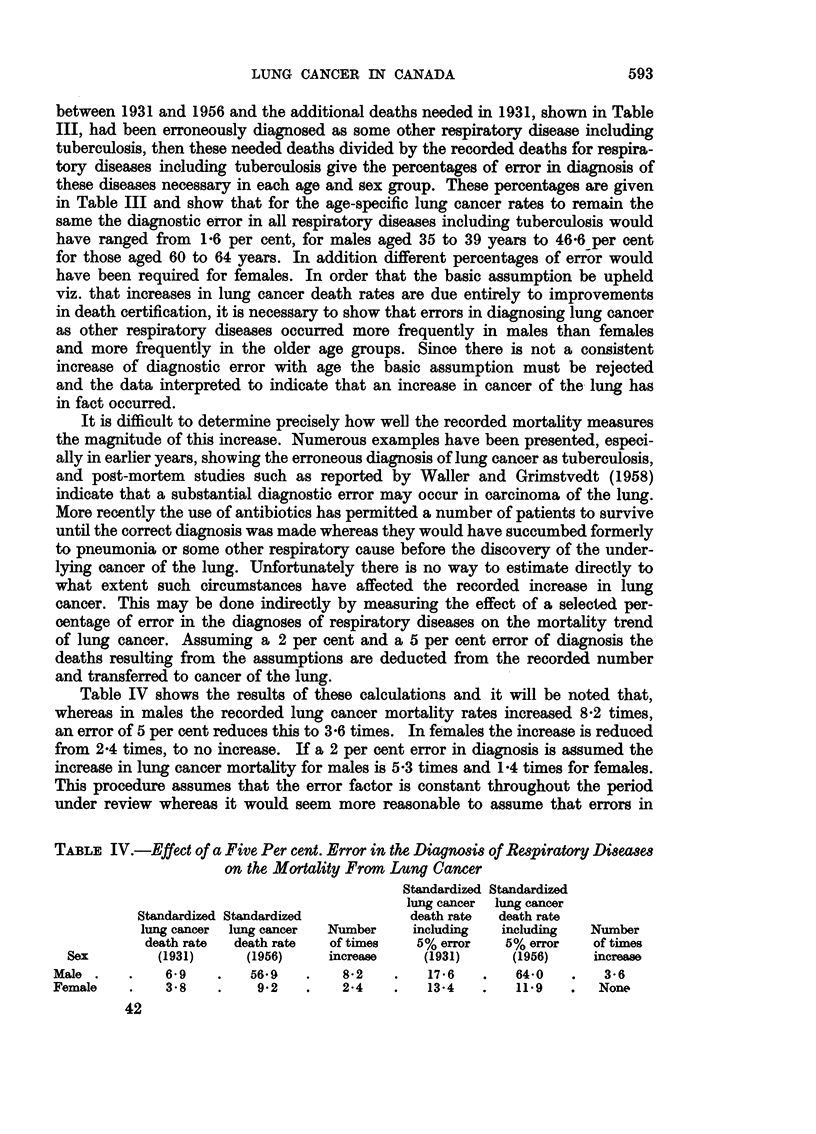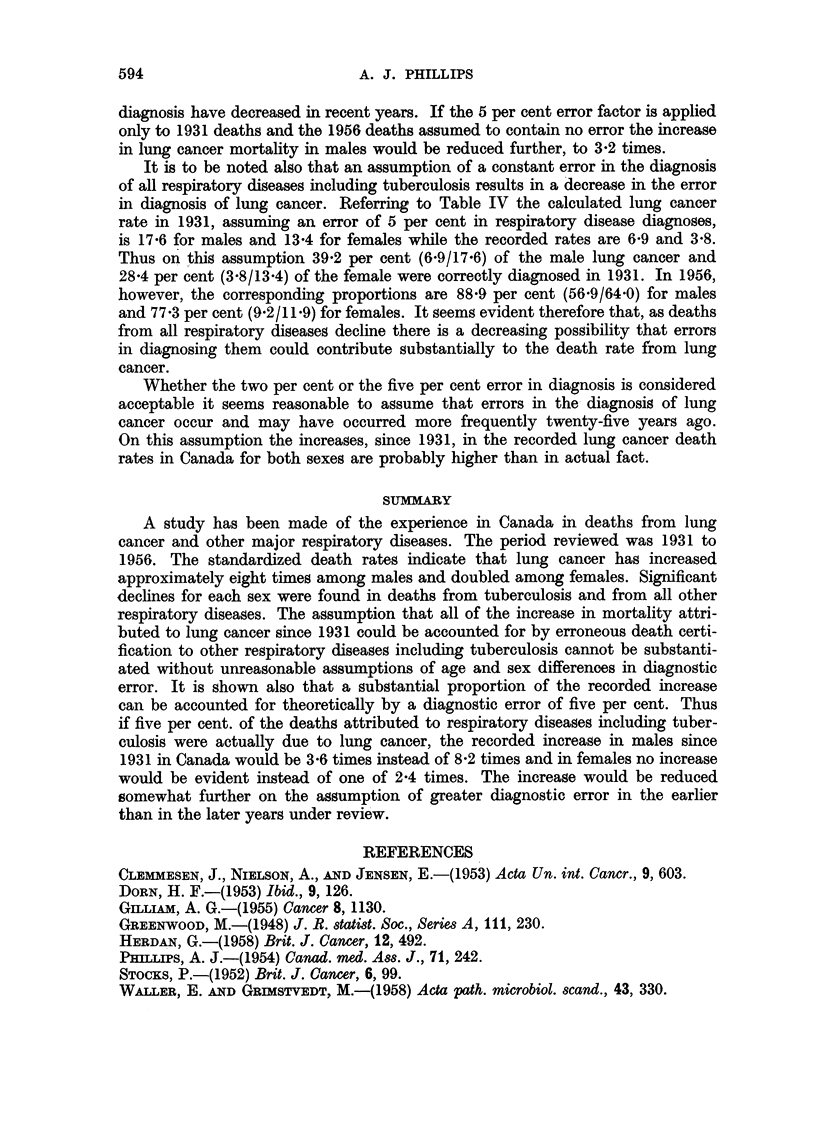# The Increase in Lung Cancer Mortality in Canada

**DOI:** 10.1038/bjc.1959.63

**Published:** 1959-12

**Authors:** A. J. Phillips


### THE INCREASE IN LUNG CANCER MORTALITY IN CANADA

A. J. PHILLIPS

*From the National Cancer Institute of Canada, 800 Bay Street, Toronto 5, Ontario, Canada*

Received for publication September 7, 1959

IN a previous paper (Phillips, 1954) it was shown that in Canada between 1931 and 1952 deaths from lung cancer had increased approximately six times among males and had more than doubled among females. The experience in numerous other countries in lung cancer mortality has been reported (Clemmesen, Nielson and Jensen, 1953 ; Dorn, 1953 ; Stocks, 1952) and equal or greater increases shown. Such studies are based, generally, upon official mortality statistics. However, in a disease which, for its correct identification, requires medical skills and facilities that are only recently coming into widespread use, the assessment of increases in lung cancer mortality requires a careful study of the relative accuracy of death certificates and special consideration for the predominant pulmonary diseases of the period under review.

Greenwood (1948) has stated that a change of fashion in death certification due to increasing knowledge may affect comparability and more recently Gilliam (1955) has written that, in lung cancer, although a large majority of informed opinion accepts the belief that the increase being experienced in many countries is real, considerable divergence of opinion remains with regard to its magnitude. Herdan (1958) has suggested that, when examining striking changes in the incidence of non-epidemic disease one must be mindful of the possibility of a change in diagnosis. In order to study such comments as they apply to mortality data in Canada a review has been made of deaths attributed to the major diseases of the respiratory system for the period 1931–1956. The sexes have been analyzed separately between the ages of 35 and 84 years. All mortality rates have been standardized on the 1951 census population of Canada in order to permit direct comparisons. The following causes of death with the International List Number were selected for study :—

| | |
|---|---|
| I. Malignant neoplasms of trachea, bronchus and lung not specified as secondary— | |
| 47 (a), (b), (c), (d) | 4th Revision |
| 47 (a), (b), (c), (d), (e), (f) | 5th Revision |
| 162, 163 | 6th Revision |
| II. Tuberculosis of the respiratory system— | |
| 23 | 4th Revision |
| 13 | 5th Revision |
| 0001 to 0008 | 6th Revision |
| III. Diseases of the respiratory system— | |
| 104 to 114 | 4th Revision |
| 104 to 114 | 5th Revision |
| A 87 to 97 (Intermediate List) | 6th Revision |
| III. (a). Bronchopneumonia— | |
| 107 | 4th Revision |
| 107 | 5th Revision |
| 491 | 6th Revision |

III. (b). Lobar pneumonia—
108 . . . . . . . . . . 4th Revision
108 . . . . . . . . . . 5th Revision
490 . . . . . . . . . . 6th Revision

III. (c). Pneumonia (unspecified)—
109 . . . . . . . . . . 4th Revision
109 . . . . . . . . . . 5th Revision
492, 493 . . . . . . . . . . 6th Revision

The standardized death rates are shown for males in Table I and Figure 1 and for females in Table II and Fig. 2. From the tables it will be noted that lung cancer mortality in males has increased from 6·9 per 100,000 population in 1931 to 56·9 in 1956, an increase of 8·2 times, while the female rates have increased approximately 2·4 times. On the other hand the tuberculosis rates have decreased from 80·8 per 100,000 to 19·2 for males and from 64·2 to 8·7 for females. All other respiratory diseases have decreased for males from 138·2 per 100,000 to 83·4 and for females from 125·7 to 44·0 per 100,000.

TABLE I.—*Standardized Death Rates Per* 100,000 *Population for Selected Causes Among Males* 35 *to* 84 *Years* (1931–1956)

| Years | Lung cancer | T.B. | All respiratory | Pneumonia: Broncho | Pneumonia: Lobar | Pneumonia: Unspecified |
|---|---|---|---|---|---|---|
| 1931 | 6·9 | 80·8 | 138·2 | 34·4 | 42·0 | 22·3 |
| 1932 | 7·4 | 75·5 | 138·0 | 36·9 | 45·7 | 22·6 |
| 1933 | 8·8 | 72·1 | 135·8 | 36·9 | 42·0 | 18·5 |
| 1934 | 8·6 | 72·2 | 135·0 | 35·7 | 45·0 | 15·0 |
| 1935 | 10·7 | 73·9 | 141·8 | 40·2 | 53·0 | 15·1 |
| 1936 | 10·9 | 72·9 | 144·6 | 44·3 | 52·8 | 14·7 |
| 1937 | 12·1 | 70·6 | 148·0 | 44·3 | 52·6 | 13·5 |
| 1938 | 12·4 | 65·1 | 140·6 | 40·2 | 54·0 | 13·0 |
| 1939 | 15·5 | 64·7 | 122·0 | 41·7 | 36·7 | 11·6 |
| 1940 | 17·3 | 64·4 | 114·5 | 36·7 | 32·9 | 9·9 |
| 1941 | 17·6 | 69·6 | 93·0 | 29·4 | 24·1 | 11·1 |
| 1942 | 16·9 | 66·1 | 90·4 | 30·3 | 25·2 | 10·7 |
| 1943 | 18·7 | 69·2 | 101·8 | 31·2 | 30·7 | 11·9 |
| 1944 | 18·5 | 63·4 | 91·8 | 28·6 | 26·2 | 11·7 |
| 1945 | 21·3 | 61·6 | 81·8 | 24·8 | 22·8 | 10·7 |
| 1946 | 24·4 | 61·2 | 84·8 | 27·5 | 19·6 | 10·7 |
| 1947 | 28·4 | 64·9 | 78·7 | 27·5 | 18·5 | 9·1 |
| 1948 | 29·5 | 57·6 | 78·5 | 27·7 | 18·8 | 9·0 |
| 1949 | 32·9 | 52·4 | 80·3 | 26·3 | 17·5 | 10·6 |
| 1950 | 36·9 | 46·4 | 73·3 | 20·2 | 12·2 | 8·0 |
| 1951 | 38·7 | 43·9 | 96·0 | 24·3 | 10·9 | 9·8 |
| 1952 | 43·0 | 33·4 | 68·4 | 22·0 | 8·1 | 7·1 |
| 1953 | 47·0 | 25·4 | 78·0 | 21·8 | 10·3 | 8·9 |
| 1954 | 50·1 | 24·0 | 66·4 | 20·7 | 8·3 | 8·2 |
| 1955 | 53·6 | 20·6 | 74·9 | 25·7 | 7·5 | 8·2 |
| 1956 | 56·9 | 19·2 | 83·4 | 27·2 | 10·3 | 7·9 |

The comments of Greenwood and Herdan regarding a change of fashion or a change of diagnosis in lung cancer death certification may be investigated by a study of the assumption that there has been no real increase in lung cancer mortality in Canada and that increases in death rates are due entirely to improvements in death certification. If this be true then following the proposal of Gilliam (1955), the number of deaths presumably resulting from cancer of the lung in 1931 may be calculated by applying the 1956 age-specific rates for each sex to

FIG. 1.—Standardized death rates for selected causes among males 35 to 84 years.

FIG. 2.—Standardized death rates for selected causes among females 35 to 84 years.

TABLE II.—*Standardized Death Rates Per* 100,000 *Population for Selected Causes Among Females* 35 *to* 84 *Years* (1931–1956)

| Years | Lung cancer | T.B. | All respiratory | Pneumonia | | |
|---|---|---|---|---|---|---|
| | | | | Broncho | Lobar | Unspecified |
| 1931 | 3·8 | 64·2 | 125·7 | 38·4 | 33·6 | 22·1 |
| 1932 | 3·9 | 60·5 | 131·6 | 38·0 | 41·5 | 24·3 |
| 1933 | 5·0 | 59·6 | 123·8 | 39·9 | 35·5 | 18·2 |
| 1934 | 4·8 | 53·5 | 121·9 | 37·3 | 36·8 | 16·9 |
| 1935 | 5·4 | 52·9 | 124·8 | 41·8 | 41·1 | 15·0 |
| 1936 | 6·1 | 51·3 | 124·9 | 38·4 | 39·6 | 14·1 |
| 1937 | 5·6 | 54·6 | 120·0 | 42·5 | 41·6 | 12·0 |
| 1938 | 5·9 | 47·2 | 112·7 | 40·3 | 37·0 | 12·0 |
| 1939 | 5·2 | 46·7 | 107·3 | 38·6 | 31·8 | 11·1 |
| 1940 | 6·2 | 42·3 | 89·4 | 34·1 | 21·9 | 9·1 |
| 1941 | 6·2 | 43·2 | 71·6 | 23·3 | 18·5 | 10·1 |
| 1942 | 6·1 | 41·9 | 68·0 | 24·1 | 15·8 | 9·6 |
| 1943 | 7·4 | 42·5 | 79·7 | 27·6 | 19·6 | 10·6 |
| 1944 | 7·5 | 43·0 | 68·1 | 23·6 | 18·5 | 9·6 |
| 1945 | 7·0 | 37·4 | 58·9 | 21·0 | 14·2 | 7·7 |
| 1946 | 7·0 | 36·5 | 60·4 | 20·8 | 13·4 | 10·0 |
| 1947 | 7·4 | 31·8 | 57·0 | 22·5 | 12·0 | 7·8 |
| 1948 | 7·9 | 31·1 | 53·2 | 20·5 | 11·6 | 5·8 |
| 1949 | 9·5 | 29·3 | 52·8 | 20·0 | 10·0 | 8·3 |
| 1950 | 7·5 | 23·6 | 52·1 | 16·8 | 7·2 | 7·7 |
| 1951 | 8·2 | 22·5 | 72·4 | 19·2 | 5·7 | 7·0 |
| 1952 | 9·2 | 14·6 | 41·0 | 14·0 | 4·7 | 5·8 |
| 1953 | 8·5 | 11·1 | 47·5 | 16·5 | 4·8 | 5·2 |
| 1954 | 8·9 | 10·0 | 38·5 | 14·4 | 4·5 | 5·4 |
| 1955 | 10·8 | 9·8 | 43·0 | 16·2 | 4·3 | 5·4 |
| 1956 | 9·2 | 8·7 | 44·0 | 16·8 | 4·3 | 7·0 |

the appropriate 1931 populations. The results so obtained, less the deaths actually recorded in 1931 give the deaths from cancer of the lung that on this assumption were, in 1931, incorrectly attributed to some other disease. These are given for males and females in Table III.

TABLE III.—*Additional Deaths from Lung Cancer Needed in* 1931* *Shown as Percentages of Deaths from all Respiratory Diseases Including Tuberculosis*

| Age Group | Additional lung cancer deaths needed in 1931 | | Additional lung cancer deaths as proportion of deaths from all respiratory diseases | |
|---|---|---|---|---|
| | Male | Female | Male (%) | Female (%) |
| 35–39 | 6 | 2 | 1·6 | 5·3 |
| 40–44 | 20 | 0 | 4·8 | 0·0 |
| 45–49 | 40 | 5 | 9·2 | 2·0 |
| 50–54 | 95 | 6 | 22·0 | 2·9 |
| 55–59 | 140 | 14 | 39·0 | 7·0 |
| 60–64 | 158 | 6 | 46·6 | 2·6 |
| 65–69 | 165 | 13 | 41·5 | 4·3 |
| 70–74 | 123 | 16 | 30·1 | 4·1 |
| 75–79 | 59 | 14 | 17·0 | 3·7 |
| 80–84 | 31 | 5 | 10·8 | 1·5 |

* In order that the 1931 and 1956 lung cancer mortality rates be equal.

It would seem reasonable to assume that deaths from cancer of the lung might be confused more frequently with tuberculosis and other respiratory diseases, than with any other cause. If there had been no increase in lung cancer deaths between 1931 and 1956 and the additional deaths needed in 1931, shown in Table III, had been erroneously diagnosed as some other respiratory disease including tuberculosis, then these needed deaths divided by the recorded deaths for respiratory diseases including tuberculosis give the percentages of error in diagnosis of these diseases necessary in each age and sex group. These percentages are given in Table III and show that for the age-specific lung cancer rates to remain the same the diagnostic error in all respiratory diseases including tuberculosis would have ranged from 1·6 per cent, for males aged 35 to 39 years to 46·6 per cent for those aged 60 to 64 years. In addition different percentages of error would have been required for females. In order that the basic assumption be upheld viz. that increases in lung cancer death rates are due entirely to improvements in death certification, it is necessary to show that errors in diagnosing lung cancer as other respiratory diseases occurred more frequently in males than females and more frequently in the older age groups. Since there is not a consistent increase of diagnostic error with age the basic assumption must be rejected and the data interpreted to indicate that an increase in cancer of the lung has in fact occurred.

It is difficult to determine precisely how well the recorded mortality measures the magnitude of this increase. Numerous examples have been presented, especially in earlier years, showing the erroneous diagnosis of lung cancer as tuberculosis, and post-mortem studies such as reported by Waller and Grimstvedt (1958) indicate that a substantial diagnostic error may occur in carcinoma of the lung. More recently the use of antibiotics has permitted a number of patients to survive until the correct diagnosis was made whereas they would have succumbed formerly to pneumonia or some other respiratory cause before the discovery of the underlying cancer of the lung. Unfortunately there is no way to estimate directly to what extent such circumstances have affected the recorded increase in lung cancer. This may be done indirectly by measuring the effect of a selected percentage of error in the diagnoses of respiratory diseases on the mortality trend of lung cancer. Assuming a 2 per cent and a 5 per cent error of diagnosis the deaths resulting from the assumptions are deducted from the recorded number and transferred to cancer of the lung.

Table IV shows the results of these calculations and it will be noted that, whereas in males the recorded lung cancer mortality rates increased 8·2 times, an error of 5 per cent reduces this to 3·6 times. In females the increase is reduced from 2·4 times, to no increase. If a 2 per cent error in diagnosis is assumed the increase in lung cancer mortality for males is 5·3 times and 1·4 times for females. This procedure assumes that the error factor is constant throughout the period under review whereas it would seem more reasonable to assume that errors in

TABLE IV.—*Effect of a Five Per cent. Error in the Diagnosis of Respiratory Diseases on the Mortality From Lung Cancer*

| Sex | Standardized lung cancer death rate (1931) | Standardized lung cancer death rate (1956) | Number of times increase | Standardized lung cancer death rate including 5% error (1931) | Standardized lung cancer death rate including 5% error (1956) | Number of times increase |
|---|---|---|---|---|---|---|
| Male | 6·9 | 56·9 | 8·2 | 17·6 | 64·0 | 3·6 |
| Female | 3·8 | 9·2 | 2·4 | 13·4 | 11·9 | None |

diagnosis have decreased in recent years. If the 5 per cent error factor is applied only to 1931 deaths and the 1956 deaths assumed to contain no error the increase in lung cancer mortality in males would be reduced further, to 3·2 times.

It is to be noted also that an assumption of a constant error in the diagnosis of all respiratory diseases including tuberculosis results in a decrease in the error in diagnosis of lung cancer. Referring to Table IV the calculated lung cancer rate in 1931, assuming an error of 5 per cent in respiratory disease diagnoses, is 17·6 for males and 13·4 for females while the recorded rates are 6·9 and 3·8. Thus on this assumption 39·2 per cent (6·9/17·6) of the male lung cancer and 28·4 per cent (3·8/13·4) of the female were correctly diagnosed in 1931. In 1956, however, the corresponding proportions are 88·9 per cent (56·9/64·0) for males and 77·3 per cent (9·2/11·9) for females. It seems evident therefore that, as deaths from all respiratory diseases decline there is a decreasing possibility that errors in diagnosing them could contribute substantially to the death rate from lung cancer.

Whether the two per cent or the five per cent error in diagnosis is considered acceptable it seems reasonable to assume that errors in the diagnosis of lung cancer occur and may have occurred more frequently twenty-five years ago. On this assumption the increases, since 1931, in the recorded lung cancer death rates in Canada for both sexes are probably higher than in actual fact.

### SUMMARY

A study has been made of the experience in Canada in deaths from lung cancer and other major respiratory diseases. The period reviewed was 1931 to 1956. The standardized death rates indicate that lung cancer has increased approximately eight times among males and doubled among females. Significant declines for each sex were found in deaths from tuberculosis and from all other respiratory diseases. The assumption that all of the increase in mortality attributed to lung cancer since 1931 could be accounted for by erroneous death certification to other respiratory diseases including tuberculosis cannot be substantiated without unreasonable assumptions of age and sex differences in diagnostic error. It is shown also that a substantial proportion of the recorded increase can be accounted for theoretically by a diagnostic error of five per cent. Thus if five per cent. of the deaths attributed to respiratory diseases including tuberculosis were actually due to lung cancer, the recorded increase in males since 1931 in Canada would be 3·6 times instead of 8·2 times and in females no increase would be evident instead of one of 2·4 times. The increase would be reduced somewhat further on the assumption of greater diagnostic error in the earlier than in the later years under review.

### REFERENCES

Clemmesen, J., Nielson, A., and Jensen, E.—(1953) *Acta Un. int. Cancr.*, **9**, 603.
Dorn, H. F.—(1953) *Ibid.*, **9**, 126.
Gilliam, A. G.—(1955) *Cancer* **8**, 1130.
Greenwood, M.—(1948) *J. R. statist. Soc., Series A*, **111**, 230.
Herdan, G.—(1958) *Brit. J. Cancer*, **12**, 492.
Phillips, A. J.—(1954) *Canad. med. Ass. J.*, **71**, 242.
Stocks, P.—(1952) *Brit. J. Cancer*, **6**, 99.
Waller, E. and Grimstvedt, M.—(1958) *Acta path. microbiol. scand.*, **43**, 330.